# Pathways towards scaling up Problem Management Plus in Turkey: a theory of change workshop

**DOI:** 10.1186/s13031-020-00278-w

**Published:** 2020-05-04

**Authors:** Daniela C. Fuhr, Ceren Acarturk, Ersin Uygun, Michael McGrath, Zeynep Ilkkursun, Sadaf Kaykha, Egbert Sondorp, Marit Sijbrandij, Peter Ventevogel, Pim Cuijpers, Bayard Roberts, on behalf of the STRENGTHS consortium

**Affiliations:** 1 Department of Health Services Research and Policy, London School of Hygiene and Tropical Medicine; Public Health and Policy, 15-17 Tavistock Place, London, UK; 2 Department of Psychology, Koc University Istanbul, Istanbul, Turkey; 3 Refugee Mental Health Branch Outpatient Clinic of Bakirkoy Mental Health Training and Research Hospital, University of Health Sciences, Istanbul, Turkey; 4 KIT Royal Tropical Institute, Amsterdam, The Netherlands; 5 War Trauma Foundation, Amsterdam, The Netherlands; 6 Department of Clinical, Neuro and Developmental Psychology, Amsterdam Public Health research institute, Vrije Universiteit Amsterdam, Amsterdam, The Netherlands; 7 Public Health Section, United Nations High Commissioner for Refugees, Geneva, Switzerland

**Keywords:** Scaling up, Theory of change, Mental health, Refugees, Low-intensity psychological intervention

## Abstract

**Background:**

A considerable evidence base has been produced in recent years highlighting the effectiveness of brief scalable psychological interventions for people living in communities exposed to adversity. However, practical guidance on how to scale up these interventions to wider populations does not exist. In this paper we report on the use of Theory of Change (ToC) to plan the scale up of the World Health Organization’s flagship low intensity psychological intervention “Problem Management Plus” (PM+) for Syrian refugees in Turkey.

**Methods:**

We conducted a one-day ToC workshop in Istanbul. ToC is a participatory planning process used in the development, implementation and evaluation of projects. It is similar to driver diagrams or logic models in that it offers a tool to visually present the components needed to reach a desired long-term outcome or impact. The overall aim of ToC is to understand the change process of a complex intervention and to map out causal pathways through which an intervention or strategy has an effect.

**Results:**

Twenty-four stakeholders (including governmental officials, mental health providers, officials from international/national non-governmental organisations, conflict and health researchers) participated in the ToC workshop. A ToC map was produced identifying three key elements of scaling up (the resource team; the innovation and the health system; and the user organisation) which are represented in three distinct causal pathways. Context-specific barriers related to the health system and the political environment were identified, and possible strategies for overcoming these challenges were suggested.

**Conclusion:**

ToC is a valuable methodology to develop an integrated framework for scaling up. The results highlight that the scaling up of PM+ for Syrian refugees in Turkey needs careful planning and investment from different stakeholders at the national level. Our paper provides a theoretical foundation of the scaling up of PM+, and exemplifies for the first time the use of ToC in planning the scaling up of an evidence-based psychological intervention in global mental health.

## Background

A large number of Syrian refugees have sought refuge in Turkey since the onset of the war in Syria in 2011. Turkey now hosts around 3.6 million Syrian refugees and ranks first as host country for Syrian refugees in terms of its numbers [1, 2]. The majority of Syrian refugees live outside camps in economically deprived urban areas across Turkey [2, 3], while around 300,000 live in camps on the Syrian border [4].

Refugees are often vulnerable to situational forms of psychosocial distress as a consequence of exposure to war and violence, potentially traumatic events experienced during the individual’s flight from their home country, and exposure to ongoing daily stressors in their new areas of settlement, such as impoverishment, unemployment, poor living conditions, social isolation and discrimination [5]. Some forms of distress may be situational while others may be more profound and can manifest in post-traumatic stress disorder (PTSD), depression and/or anxiety disorder [6]. Currently, there are no population wide estimates on the prevalence of mental disorders among refugees in Turkey. Acarturk et al. [7] investigated the prevalence of probable PTSD and depression among adult Syrians residing in a camp near the Syrian / Turkish border, and reported that around 83% screened positive for PTSD while around 37% screened positive for symptoms of depression. In a cross-sectional study conducted in a tent city in Gaziantep, Turkey, Alpak et al. reported a PTSD prevalence of 33.5% among Syrian refugees [8]. Data from our own cross-sectional survey of Syrian refugees in Sultanbeyli, Istanbul revealed a prevalence of symptoms of PTSD, depression and anxiety of 19.6, 34.7 and 36.1% respectively [9]. Variability of prevalence estimates may result from differences in the conditions in which the respondents were living, and methodological differences between the surveys [5].

Mental health services in Turkey are overseen by the Turkish Government’s Ministry of Health [10]. A national mental health action plan was developed in 2011 [11]. However, budget limitations have hampered the integration of mental health into primary and community care, with most care still delivered by psychiatrists, psychologists and other mental health professionals at the tertiary and secondary care level [12]. This form of treatment might be beneficial for more serious cases of mental disorders, and for Turkish residents as treatment is delivered in Turkish. Registered Syrian refugees can formally access the public mental health care health system in Turkey but need to speak Turkish or have an interpreter available in order to benefit from treatment. Structural and attitudinal barriers to accessing the public health care system have been reported for refugees, resulting in unmet need and a large mental health treatment gap for Syrian refugees in Turkey [9, 13]. Culturally and linguistically sensitive health services are provided to Syrian refugees through 178 refugee health centres established as part of the WHO Refugee Health Programme [14]. These centres are not part of the formal public health care system but are community centres where Syrian doctors provide care for Syrian patients [15]; these centres also serve as gateways to health care for Syrian refugees [14]. There is also a range of nongovernmental organisations (NGOs) involved in provision of mental health and psychosocial support activities for Syrian refugees in Turkey [16, 17]. However, there remains a need for evidence-based, community-based interventions for Syrian refugees in Turkey which addresses Syrian refugees’ mental health needs in a culturally relevant and scalable way.

### Problem management plus (PM+) in Turkey

Problem Management Plus (PM+) was designed by the World Health Organization (WHO) for adults impaired by distress in communities exposed to adversity [18–21], and is currently being adapted for Syrian refugees residing in countries neighbouring Syria, including Turkey [22]. PM+ is a transdiagnostic intervention (i.e., not condition-specific) to reduce common mental health symptoms such as anxiety, depression and posttraumatic stress and to improve psychosocial functioning. PM+ is a 5-session intervention, comprised of evidence-based techniques for problem solving, stress management, behavioural activation, and accessing social support [19]. In South Turkey, the WHO organized ‘trainings for trainers’ in PM+ for Syrian mental health professionals who subsequently trained psychosocial workers providing individual PM+ for Syrians in North East Syria and South Turkey. In Sultanbeyli/Istanbul, PM+ is provided to Syrian refugees in a group setting. Group PM+ providers are female and male peer-refugees with a background in health care, social work or community care who receive 8 days of training, followed by three practice cases, on-the-job training, and close supervision during implementation delivery. PM+ trainers/supervisors are licensed mental health care professionals such as psychologists or psychiatrists.

### Objective of this paper

The last decade has seen a rise in the development and evaluation of low-intensity psychological interventions [23]. Many have proven effectiveness for improving mild to moderate mental health symptoms; however, population-level coverage remains low, due to a range of implementation challenges related to limited adoption in policies and strategies, insufficient resource allocation, competing national interests, and a lack of planning and guidance regarding how to take psychological interventions to scale [24, 25]. In this paper we test the use of Theory of Change (ToC) to plan the scaling up of a low-intensity psychological intervention. ToC is a participatory planning process used in the development, implementation and evaluation of projects [26]. To the best of our knowledge, ToC has not been applied to scaling up public health interventions yet. The aim of this paper is to present the ToC map for scaling up group PM+ in Turkey. Our objectives were to (a) investigate the use of ToC methodology in planning the scale up of PM+ for Syrian refugees in Turkey; (b) to explore context-specific pathways of scaling up PM+ for Syrian refugees in Turkey; and (c) to identify barriers and facilitators to scale up.

## Methods

We conducted a one-day ToC workshop on 8 November 2018 in Istanbul, Turkey. Twenty-four stakeholders participated in the workshop (10 national and international academics and mental health/conflict researchers from universities in Turkey, the United Kingdom and the Netherlands; 10 staff from national and international NGOs such as UNHCR, Relief International Turkey, War Trauma Foundation, International Blue Crescent; three psychiatrists and psychologists from local hospitals and community centres, and one government official from the Ministry of Health in Ankara). At the beginning of the workshop, PM+ was introduced to external stakeholders who were not involved in developing and adapting PM+ in Turkey. This was followed by a presentation of results from formative research and the PM+ pilot trial in Turkey. A short introduction to scaling up innovations and the concept of ToC was provided to participants. The ToC workshop and the development of the ToC map was informed by the ExpandNet framework of scaling up health service innovations [25, 27, 28].

### Scaling up interventions

The literature offers a number of frameworks and theories of how interventions can be taken to scale [25, 27–34]. Perhaps the most comprehensive framework and systematic approach for implementers is the WHO ExpandNet framework of scaling up [25, 27] which understands scaling up as “*deliberate efforts to increase the impact of health service innovations successfully tested in pilot or experimental projects so as to benefit more people and to foster policy and programme development on a lasting basis*” [35]. Compared to other frameworks, the WHO ExpandNet framework elaborates on the necessary elements of scaling up and the attributes of success [25], and offers practical guidance on how interventions can be taken to scale [28]. The WHO ExpandNet framework understands scaling up as an open system of five elements: (1) the innovation, (2) the resource organisation or resource team, (3) the user organisation, (4) the environment, and (5) the scaling up strategy. The innovation refers to the intervention which is being scaled up. The resource team provides guidance and technical assistance to the deliberate efforts to utilise the innovation at scale. The resource team can include different stakeholders such as researchers but also personnel from the organisation that seeks to adopt the innovation such as governmental officials. The user organisation refers to the institutions or organisations that are expected to adopt and implement the innovation at scale, such as the public health system, NGOs, the private services or any combination of other services or institutions. The WHO ExpandNet framework defines the environment as external barriers or facilitators which can promote or hamper the scale up, such as local or national policies, bureaucratic structures, the health sector, socio-economic or cultural constraints, as well as people’s needs and rights. Finally, the scaling up strategy is understood as plans and actions for scaling up including the means by which the innovation is communicated, disseminated, transferred or promoted [36]. The WHO ExpandNet framework suggests that scaling up of an intervention should be planned through a participatory process with key stakeholders [25, 27], however, it does not suggest a theory or methodology of how to do this.

### Theory of change

ToC has been used in global mental health, specifically during formative research to conceptualise the delivery of mental health programmes [37, 38] but also to plan the implementation of mental health care plans and services [39, 40]. ToC is similar to driver diagrams or logic models in that it offers a tool to visually present the components needed to reach a desired long-term outcome or impact. However, in contrast to driver diagrams or logical models, it allows feedback loops and shows how different pre-conditions interact with each other [38]. The overall aim of ToC is to understand the change process of a project and to map out causal pathways by presenting the sufficient preconditions (called “*intermediate outcomes”* for the remainder of this paper) which lead to the desired “*long-term outcome”* or envisaged “*impact”* the project intends to achieve [26]. Long term outcomes are the final and measurable outcomes that the project can achieve on its own, whereas impact refers to the change or real-world impact the project envisages to contribute towards [38]. The impact is behind the ceiling of accountability: the level at which implementers stop measuring whether outcomes of the project have been achieved, and therefore stop accepting responsibility [26, 41]. ToC requires stakeholders to think about “*assumptions”* and “*interventions”* as well. Assumptions are external conditions which must exist for the intermediate outcome on the causal pathway to be achieved, whereas interventions are strategies or activities that bring about intermediate outcomes [26, 38, 41].

The causal pathways of scaling up PM+ for Syrian refugees in Turkey was developed together with stakeholders in our one-day participatory workshop, and was further contextualised and finalised afterwards through small group discussions with Turkish researchers and mental health professionals. Assumptions and interventions of the ToC map were informed by the results of the formative research and pilot phase in Turkey as well as qualitative data assessing the responsiveness of the Turkish mental health system. These data will be published elsewhere.

## Results

**Fig. 1:**
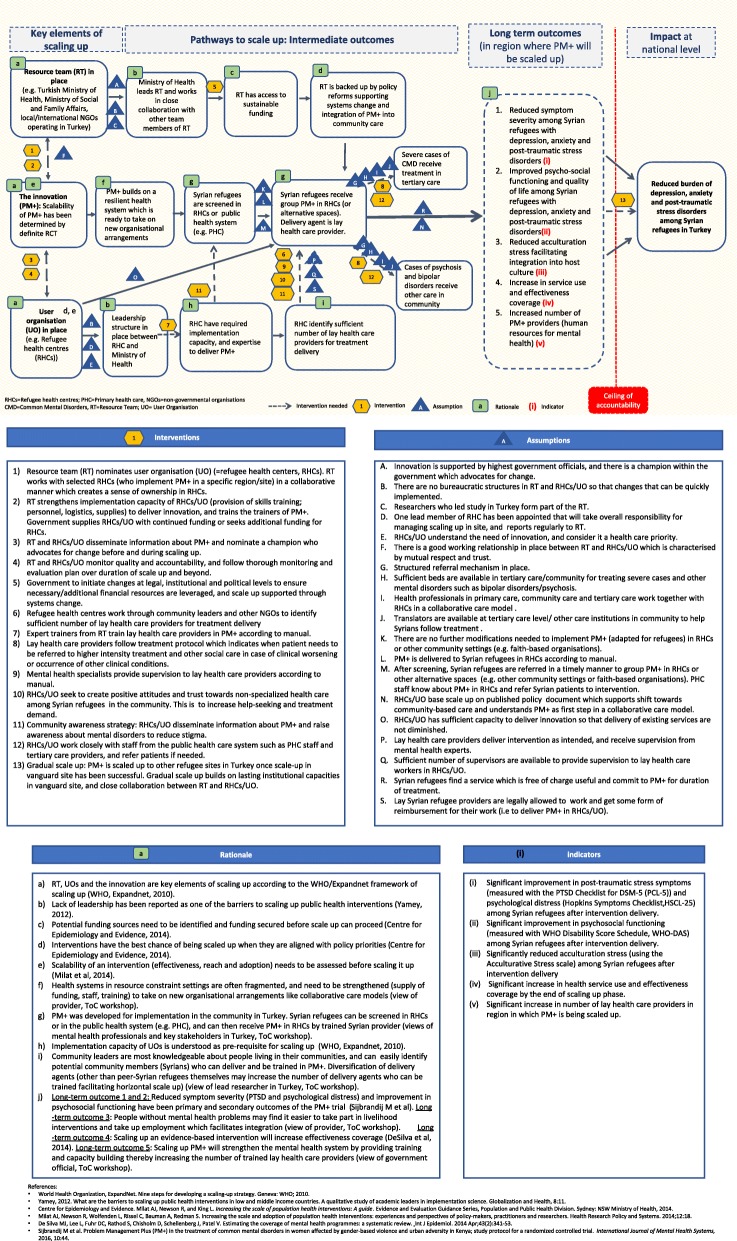
ToC map for planning the scale up of group-based PM+ in Turkey

The ToC map is presented in Fig. 1 alongside a legend describing interventions, assumptions, rationale, and indicators. The ToC map should be read from left to right. Three key elements of scaling up were identified (the resource team; the innovation and the health system; and the user organisation) which are represented in three distinct causal pathways. Thirteen interventions (intervention 1–13) and 20 assumptions (assumption A-T) were identified by stakeholders. In addition, intermediate outcomes were supported by 10 rationales (rationale a-j). Key assumptions and interventions are included in the description of the causal pathways further below. Please refer to the legend for the complete list of assumptions, interventions and rationales.

### Pathways to scale up

The three pathways to scale up led to five long-term outcomes and an envisaged impact (shown on the right-hand side of the ToC map). Stakeholders identified “Reduced burden of psychological distress and reduced symptoms of depression, anxiety and PTSD among Syrian refugees in Turkey” as the vision or impact that the scaling up of group PM+ may be able to contribute towards. Long term outcomes apply to the population and the health system in the district/region in which PM+ is being scaled up. Five long term outcomes were identified: Reduced symptom severity among Syrian refugees; improved psychosocial functioning and quality of life among Syrian refugees; reduced acculturation stress of Syrian refugees facilitating integration into host communities; increase in service use and effectiveness coverage; and increased number of human resources for mental health. Indicators have been developed for these long-term outcomes which can be used to measure success of the scale up strategy in the region where it will be scaled up. These indicators are outlined under Fig. 1 (see box “indicators”).

#### Resource team pathway

The resource team was perceived as an important pillar of the scaling up strategy. ToC workshop participants argued that the resource team should comprise of the Ministry of Health, Ministry of Social and Family Affairs, key NGOs and the Turkish researchers (see assumption C) who developed and adapted PM+ in Turkey. An early intermediate outcome to the resource team’s pathway is leadership within the resource team. Leadership was thought to be provided by the Ministry of Health or other governmental bodies that have the necessary political power to bring about sustainable funding. It was further argued that sustainable funding should be based upon policy documents outlining reforms for system’s change. A key assumption on this pathway was that the innovation must be supported by senior government officials, and that there is a champion within the government who advocates for change (assumption A). For scale up to happen stakeholders perceived a need for the government to initiate changes at the legal, institutional and political levels to ensure additional financial resources are leveraged (intervention 5).

#### Innovation pathway

The second pathway to scale up is the innovation pathway and focuses on the PM+ intervention itself. It understands scalability of PM+ (i.e. effectiveness of PM+, its wider population reach, and adoption) as an essential pre-requisite before PM+ can be rolled out. Stakeholders noted that PM+ should build on a resilient health system. For successful integration, PM+ should be nested in a health service structure which is functioning well and able to assimilate new organisational arrangements like collaborative stepped care. In a collaborative stepped care model, PM+ would be understood as first treatment step for mild or moderate mental disorders. Due to the health service structure in Turkey, and the need to deliver interventions to Syrian refugees in a cultural relevant way, refugee health centres were identified as preferred delivery platform for scale up. Screening was suggested to take place in either refugee health centres or primary health care while PM+ itself would be offered by Syrian lay health providers in refugee health centres only. Individuals displaying clinical worsening or serious mental disorders such as psychosis would not receive group PM+ but would be referred to tertiary care or other community health care centres for appropriate treatment. A few assumptions around the health system were underlying this pathway; for example, it was assumed that the health system and its staff are responsive to the needs of Syrian refugees and support change (assumption M); that a structured referral mechanism would be in place (assumption G); that an increase in referrals to tertiary care would be absorbed by the public health system (assumption H), and that translators would be available in secondary or tertiary care to guide Syrians through treatment (assumption J).

#### User organisation(s) pathway

The third pathway refers to the user organisation. Participants of the ToC workshop suggested refugee health centres as user organisation which should offer and implement PM+. A leadership structure between the Ministry of Health and the refugee health centres was considered essential for success. Another key requirement was for refugee health centres to have both the capacity and expertise to implement PM+. Refugee health centres were suggested to work through community leaders and NGOs to recruit Syrian lay health care providers for treatment delivery. ToC workshop participants assumed that one refugee health centre would be appointed to take overall responsibility for managing scaling up of PM+ in the site where it will be scaled up, and that this lead organisation would also report and update the resource team on progress being made (assumption D).

Finding enough Syrian lay PM+ providers was an issue discussed extensively. It was suggested that refugee health centres work with community leaders and NGOs to identify a sufficient number of lay health care providers to meet treatment demand (intervention 6). Another key assumption was the availability of mental health specialists to supervise lay providers (assumption Q), and that those delivering PM+ would get some form of reimbursement (in form of a stipend or salary) for their work (assumption S). To support the uptake of PM+, refugee health centres would need to raise community awareness about the intervention and mental disorders (assumption 11) and foster positive attitudes and trust in non-specialised health care among Syrian refugees (assumption 10).

A key intervention was suggested between the resource team and the user organisation: the resource team was thought to be responsible to strengthen implementation capacity of refugee health centers though provision of skills training, personnel and logistics to deliver PM+, and was thought to be in the best position to train the trainers of PM+ (intervention 2). Both the resource team, and the refugee health centers would be required to monitor quality and accountability of the scale up, and follow a thorough monitoring and evaluation plan (intervention 4).

## Discussion

To the best of our knowledge, this is the first paper which reports the use of ToC in planning the scale up of a public health intervention. Scaling up of evidence-based interventions is essential to overcome the mental health treatment gap. Unfortunately, we are still far from reaching that goal [24]. Evidence-based interventions to improve mental health outcomes are available; however, they need to be implementable in the community or primary health care for coverage to be expanded. Barriers to successful integration and scale up are known and include low acceptability, appropriateness, and programme credibility from patient and provider; lack of knowledge and skills of the provider; poor motivation to change (provider and health system); poor management and/or leadership; and lack of financial resources [23, 42]. Some of these barriers can be overcome during intervention development by conducting comprehensive formative research with patients, providers and key stakeholders regarding the acceptability, feasibility and likely sustainability of the intervention.

### Importance of the ToC workshop in Turkey

We found several advantages of exploring the scale up of PM+ using ToC. First, scaling up is a process which is not neutral [35], and usually involves balancing the conflicting interests of different stakeholders. ToC helped us develop an integrated framework for scaling up PM+ in Turkey by engaging with key stakeholder groups in national/local government, NGOs, and Syrian refugee health care clinics who had provided different perspective and knowledge of the local health system and socio-political context. The structured working approach of ToC and the guidance received by the ToC facilitator who was neutral to the development of the ToC map supported allowing ToC participants to discuss critical issues in an equitable way. Second, the ToC workshop also provided opportunities for participants to discuss potential health system bottlenecks, and institutional, operational and political barriers to scaling up. Facilitators to overcome some of these barriers (i.e. interventions) were then suggested. Third, the ToC map highlighted the complexity of scaling up PM+ to local stakeholders, and the importance of early planning and engagement.

A critical issue for ToC workshop participants was the platform of care where PM+ would be delivered. PM+ delivery was suggested through refugee health centres rather than NGOs or primary health care. Currently, refugee health centres receive financial support from the government and the European Union [43, 44]. Implementation through refugee health centres was thought to be more sustainable compared to implementation by NGOs as NGOs may operate on a time-limited budget. Moreover, work permission of NGOs is reviewed annually by the Turkish government. Implementation of PM+ through primary health care was also not considered feasible as Syrian doctors or nurses are not allowed to work in the public health system in Turkey [45] so that PM+ would have to be delivered in Turkish by Turkish providers. Treatment delivery by a foreign provider who does not speak the mother tongue of the patient has been found to be a barrier to mental health treatment seeking and continuation [46, 47]. Refugee health centres were therefore thought to be the most viable option. Syrian medical doctors receive training from Turkish providers before being able to work in refugee health centres, and this includes trainings with materials from the mental health Gap Action Programme (mhGAP) [48]. The mhGAP Intervention Guide recommends brief psychological treatments for depression or posttraumatic stress disorder such as PM+ for mild or moderate symptoms [48]. However, currently no evidence-based manualised psychological interventions are being offered in refugee health centres, which limits the implementation of mhGAP guidelines by Syrian providers. The implementation capacity of refugee health centres remains key and is an essential intermediate outcome on the causal pathway to scale up PM+. To address limited staff capacity, PM+ could be offered in selected refugee health centres to which Syrian refugees with mental health problems would be referred. The government would have to make an additional investment in those refugee health centres, and equip them with additional funding to support a core team working exclusively on PM+. Scaling up PM+ through refugee health centres relies on a good working relationship and collaboration with the public health system as more serious cases of mental disorders would then need to be referred to higher intensity treatment in the public health care system.

### Limitations

Our paper has a number of limitations. First, the ToC map is built upon a hypothetical scenario as the scalability of PM+ in Turkey has not yet been determined; the trial in Turkey is currently ongoing, with results expected by December 2021. Second, we developed indicators for long-term outcomes only, as the ToC map will not yet be used to monitor or evaluate the success of the scaling up pathways. We also suggest that our indicators for long-term outcomes be made more specific once a region for scaling up PM+ has been selected. These indicators should then be time-related, specifying when results be achieved. Third, we were unable to involve patient user groups in our ToC workshop. Patient user groups could have provided additional insights into the implementation of PM+ during scale-up which may not have been captured by stakeholders who were present at the workshop. However, patients have been interviewed in the formative research phase in Turkey, and findings of these qualitative interviews informed the development of the ToC map. Fourth, ToC is a methodology to map out how change occurs and outlines the sufficient and essential intermediate outcomes. It does not investigate the reasoning behind the change process itself and this could be further investigated through in-depth qualitative research. Finally, we did not discuss the scaling up strategy as such. The scaling up strategy is understood as “plans and actions for scaling up including the means by which the innovation is communicated, disseminated, transferred or promoted” [25]. Stakeholders at the government and other key stakeholders such as the ones who participated in the ToC workshop may want to discuss details of the scaling up strategy once the framework of scaling up has been finalised.

## Conclusions

Research results, such as from randomised controlled trials, are rarely sufficient to change service structures, and it can take a long time for evidence-based interventions to be implemented on a large scale [25]. We found ToC a particularly useful exercise to discuss the potential scale up of PM+ for refugees in Turkey, and will test its use for planning the scale up of PM+ in other sites in the future. Early planning and engagement of key stakeholders is essential to pave the way for scaling up an evidence-based intervention. With the help of ToC, we were able to provide a framework of scaling up PM+ which can be further adapted by stakeholders once the (cost-)effectiveness and reach of the PM+ trial in Turkey is known.

## Data Availability

Not applicable.

## Abbreviations

ToC: Theory of Change
PM +: Problem Management Plus
WHO: World Health Organization
